# Antenatal care and childbirth experience among parents of children with orofacial clefts: an observational study

**DOI:** 10.1186/s12903-026-08513-1

**Published:** 2026-05-13

**Authors:** Iffatul Amaaniyah, Anne Agustina Suwargiani, Fidya Meditia Putri

**Affiliations:** 1 Undergraduate Program, Faculty of Dentistry, Universitas Padjadjaran, Bandung, Indonesia; 2 Dental Public Health Department, Faculty of Dentistry, Universitas Padjadjaran, Bandung, Indonesia

**Keywords:** Antenatal care, Childbirth, Orofacial clefts

## Abstract

**Background:**

The experiences of antenatal care (ANC) and childbirth are crucial in identifying and managing conditions such as orofacial clefts, which are often not detected early enough, both prenatally and postnatally. This study explores the ANC and childbirth experiences of parents with children affected by orofacial clefts, aiming to highlight the role of ANC in supporting families and preparing them for long-term care plans.

**Methods:**

This descriptive observational study used a survey conducted at the *Yayasan Pembina Penderita Celah Bibir dan Langit-Langit* (YPPCBL), Bandung, between July and August 2024. A purposive sampling method based on the Cochran formula was used to select 49 respondents, with 55 ultimately participating. Data were collected primarily and analyzed descriptively via frequency distribution.

**Results:**

The study which involved 55 respondents, revealed that most mothers received ANC services (98.2%), although adherence to six recommended ANC visits (20%) and the Ten Treatments (10T) ANC standards (34.5%) were low. Prenatal diagnosis of orofacial clefts occurred in 23.6% of cases, primarily through ultrasound examinations, while the majority (76.4%) were identified postnatally. A small proportion of mothers delivered outside healthcare facilities (7.3%) or without medical assistance (3.6%). Newborn physical examinations were conducted in 81.8% of cases.

**Conclusions:**

Despite high ANC attendance, there were deficiencies in compliance with ANC K6 visits and the implementation of integrated 10T ANC standards. Prenatal detection of orofacial clefts remains rare, with most cases identified postnatally. While most births occurred in healthcare facilities, palatal clefts often went undetected due to insufficient newborn examinations. These findings underscore the need to enhance ANC services and neonatal examinations to improve early detection and management of orofacial clefts.

**Supplementary Information:**

The online version contains supplementary material available at 10.1186/s12903-026-08513-1.

## Background

The health of pregnant women and fetuses is very important to maintain during pregnancy because this period determines the development of the fetus until birth. The labor process also affects the development of children postnatally, as newborn must undergo critical physiological adaptations after birth [1]. Failure to adapt successfully may threaten survival and hinder developmental progress [1]. These adaptations can be more challenging in the presence of congenital anomalies such as orofacial cleft, underscoring the importance of early detection and delivery planning through ANC. Adequate prenatal care has been shown to reduce the severity of complications, such as Neonatal Intensive Care Unit (NICU) admission or transfers, in infants with cleft lip and/or palate [2].

Healthy pregnancies and deliveries can be monitored through regular pregnancy or antenatal care (ANC) check-ups. According to the World Health Organization (WHO), an estimated 260,000 women died from maternal causes in 2023, equivalent to more than 700 deaths per day, or approximately one every two minutes [3]. Maternal mortality and stillbirths predominantly occur in low- and middle-income countries, where most cases remains preventable [3]. In Indonesia, the national targets set by the National Medium-Term Development Plan (RPJMN) 2020–2024 are to reduce the maternal mortality ratio (MMR) to 183 per 100,000 live births and the neonatal mortality rate (NMR) to 10 per 1,000 live births [4] far According to WHO and United Nations Inter-agency Group for Child Mortality Estimation (UN IGME), Indonesia’s MMR in 2023 was 140 per 100,000 live births, and the NMR was approximately 11 per 1,000 live births [3, 5].

Antenatal care (ANC) is very important for preventing the complications of pregnancy and childbirth. On the basis of the Integrated ANC Guidelines, ANC is a comprehensive series of activities conducted from conception until delivery [4]. The WHO recommends eight ANC visits during pregnancy, whereas in Indonesia, a minimum of six visits are needed, including at least two contacts with a physician to screen for complications [4, 6]. One of the main objectives of ANC is to prevent complications associated with pregnancy and childbirth [7]. However, in Indonesia, ANC visits for pregnant women have not been a top priority for many pregnant women themselves [8].

Another significant benefit of ANC is the ability to detect congenital abnormalities such as orofacial clefts. Orofacial clefts are among the most common congenital malformations in the world, with a global prevalence of approximately 1 in 700 live births, and in Asia, approximately 1 in 500 births [9, 10]. In Indonesia, the prevalence of orofacial clefts in children aged 0–59 months is 0.24 [11]. Prenatal detection of this condition can be performed through ultrasound screening (USG), which is the gold standard [12]. Typically, these abnormalities can be detected at 20 weeks of gestation [13]. However, many cases of orofacial clefts, especially cleft palate (CPO), are diagnosed only postnatally [13, 14]. The low accuracy of prenatal detection poses a challenge, particularly in diagnosing cleft palate, which is difficult to diagnose early because of difficulties in visualization [12, 15]. Delayed diagnosis often results in delayed medical interventions, impacting the baby’s care and the family’s preparedness in dealing with this condition [16].

Prenatal diagnosis of an orofacial cleft provides valuable time for the family to prepare for the baby’s care [16]. Prenatal counseling provides an opportunity for parents to gain knowledge about their condition, long-term medical intervention plans such as surgery and speech therapy, and psychological support. This counseling also enables families to adapt psychologically, helping them manage the anxiety and stress that often arise due to a lack of preparedness [16]. Early diagnosis also helps parents undergo genetic testing to identify underlying genetic issues [16, 17]. Additionally, safe delivery planning is also possible in healthcare facilities that are capable of handling babies with special needs, ensuring safety for the mother and helping the family manage stress and anxiety [16, 17].

In addition to ANC, childbirth assisted by skilled healthcare professionals also plays a crucial role in reducing maternal and neonatal mortality rates. Factors such as the choice of delivery location and the type of birth attendant impact maternal and infant health. More than half of maternal deaths occur during childbirth, making childbirth a highly critical phase [18]. Research by Gonzalez et al. revealed a correlation between socioeconomic status and the occurrence of orofacial clefts [19]. Compared with those born in private hospitals, infants born at home or in government-funded public hospitals, often used as indicators of low economic status, had a greater risk of orofacial clefts. The use of public hospitals as healthcare facilities may reflect limited access to more expensive or higher-quality medical services, which is associated with the family’s socioeconomic status. This factor contributes to the pattern of orofacial cleft occurrence. Furthermore, birth order is also believed to influence the condition, with later-born children being more vulnerable to orofacial clefts than firstborn children are [20].

This study aims to explore the experiences of ANC and childbirth among parents of children with orofacial clefts, with a focus on early detection, delivery planning, and psychological support for families. The results of the study are expected to provide an understanding of the importance of ANC in supporting families facing the birth of a child with an orofacial cleft while encouraging family acceptance and preparedness to undergo long-term care plans. Research on the experiences of ANC and childbirth among parents of children with orofacial clefts in Indonesia has not been conducted, highlighting a research gap that needs further exploration. While some studies have examined the factors influencing ANC behaviors in Indonesia and the impact of the COVID-19 pandemic on ANC, research specifically addressing ANC experiences related to orofacial cleft cases in Indonesia is scarce [21–23]. Some studies from other countries, such as Olusanya et al.‘s study in Nigeria, have shown that although ANC is common among mothers of children with orofacial clefts, prenatal detection of the condition remains very low [24].

## Methods

### Study design and setting

This study was a descriptive observational study with a survey method. It was conducted at *Yayasan Pembina Penderita Celah Bibir dan Langit-Langit* (YPPCBL) in Bandung city from July to August 2024.

### Participants and sampling procedure

The population consisted of parents of children with orofacial clefts who visited YPPCBL during the research period. The sampling technique was performed via purposive sampling. Respondents were either the mother or father who accompanied the child to YPPCBL. Interviews were conducted with whichever parent was present and willing to provide complete information about their ANC and pregnancy history.

### Inclusion and exclusion criteria

The inclusion criterion included parents of children aged 0–8 years with orofacial clefts. Whereas the exclusion criterion included parents who were unwilling to participate [25].

### Sample size calculation

On the basis of Cochran’s formula calculations, the minimum required sample size was 49 participants [26].

$$n=\frac{{Z}^{2}pq}{{e}^{2}}$$

where:

*n* = minimum sample size

*Z *= *Z*-value (1.96 for 95% confidence level)

*p *= estimated proportion of the population (0.5 used as a conservative estimate)

*q* = 1-*p*

*e* = margin of error (0.05)

using *Z* = 1.96, *p* = 0.5, and *e* = 0.05, the minimum sample size required was 49 respondents.

### Data collection and instrument

The data collection instrument was a questionnaire designed on the basis of the Indonesian Health Survey 2023 [11]. The full English version of the questionnaire is available as Supplementary File 1. The questionnaire consists of questions regarding respondents’ sociodemographic characteristics, antenatal care (ANC), and childbirth history. Data were collected through direct interviews with respondents conducted by the researchers. Before they participated, the respondents were provided with detailed explanations of the study’s purpose and signed written consent.

### Validity and reliability testing

The research instrument was designed and through modification based on the Indonesian Health Survey 2023 questionnaire form. It underwent validity and reliability testing. Validity was assessed via the content validity method, which involves three experts to evaluate content validity (NS, IH, ANS). The validity test results indicated that all questionnaire items achieved an I-CVI score of 1.00, indicating validity, with a total content validity score (S-CVI) also reaching 1.00 [27]. Reliability was tested via the test‒retest method to assess the instrument’s internal consistency. Pearson correlation calculations resulted in a score of 0.838, which was categorized as very strong, demonstrating high instrument consistency [28].

### Statistical analysis

The collected data were analyzed descriptively using Microsoft Excel. Frequency and percentage distributions were used to summarize the characteristics of the respondents and research variables.

### Policy context and variable classification

This study also considered changes in policies related to antenatal care (ANC) visit standards applied during the respondents’ pregnancy periods. Mothers who gave birth before 2021 were categorized as receiving the ANC K4 (antenatal care with four visits) policy, which required a minimum of four visits during pregnancy.

Starting in 2021, the Indonesian government implemented the ANC K6 (antenatal care with six visits) policy, which mandates a minimum of six antenatal care visits distributed across pregnancy stages: one visit in the first trimester, two in the second trimester, and three in the third trimester. This policy aims to improve maternal and fetal health monitoring by increasing the frequency and quality of care, including at least two ultrasound examinations performed by a physician. The ANC K6 policy and visit schedule are regulated under the Indonesian Ministry of Health Regulation Number 21 of 2021 concerning the Implementation of Health Services Before Pregnancy, During Pregnancy, Childbirth, and Postpartum Period [29].

In addition to visit frequency, ANC services in Indonesia are guided by the “Sepuluh (10) Tindakan” or “Ten Treatments” standard, which ensures comprehensive maternal assessment and preventive care. This includes: (1) measuring maternal weight and height, (2) measuring blood pressure, (3) measuring upper arm circumference (LILA), (4) measuring fundal height (TFU), (5) assessing fetal presentation and monitoring fetal heart rate, (6) screening tetanus immunization status and administering Td vaccines, (7) providing iron supplementation, (8) conducting laboratory tests (including HIV, syphilis, and hepatitis B screening), (9) managing pregnancy-related conditions within the health worker’s scope of practice, and (10) delivering counseling and psychosocial support. These components are used as the reference standard to assess the comprehensiveness of ANC received by respondents [4].

Additionally, respondents’ residential locations were cross-referenced with the 2020 Urban and Rural Classification Data for Indonesia issued by the Central Statistics Agency (BPS), ensuring accurate categorization of locations as urban or rural [30] periods.

## Results

On the basis of the specified inclusion and exclusion criteria, 55 respondents participated in this study (Table 1). Most respondents completing the questionnaire were mothers (*n* = 53, 96.4%), followed by fathers (*n* = 1, 1.8%), and others, in this case, siblings of the parents (*n* = 1, 1.8%). Most mothers were aged 20–35 years during pregnancy (*n* = 46, 83.6%), had a middle education level (*n* = 25, 45.5%), and were unemployed (*n* = 41, 74,5%). Moreover, most fathers had a middle education level (*n* = 28, 50.9%) and were employed (*n* = 51, 92.7%). Geographically, the majority of families resided in urban areas (*n* = 44, 80%), whereas the remaining (*n* = 11, 20%) lived in rural areas.

**Table 1:** Characteristics of parents (*n*=55)

Variable	Number (*n*)	Precentage (%)
Parents		
Father	1	1.8
Mother	53	96.4
Other	1	1.8
Mother’s age when pregnant		
<20 years	2	3.6
20–35 years	46	83.6
>35 years	7	12.7
Father’s education		
Low	19	34.5
Medium	28	50.9
High	8	14.5
Mother’s education		
Low	18	32.7
Medium	25	45.5
High	12	21.8
Father’s Occupation		
Employed	51	92.7
Unemployed	4	7.3
Mother’s Occupation		
Employed	14	25.5
Unemployed	41	74.5
Location		
Urban	44	80
Rural	11	20

Table 2 presents the characteristics of the children with orofacial clefts from the study respondents. Most children (*n* = 39, 70.9%) had visited YPPCBL more than three times. With respect to sex, mostly (*n* = 36, 65.5%) were male, while the remaining (*n* = 19, 34.5%) were female. Most of the children were 4 years old (*n* = 13, 23.6%) or 3 years old (*n* = 12, 21.8%), with the majority were firstborn (*n* = 23, 41.8%) being firstborn. The most common type of orofacial cleft was a combination of cleft lip and palate (*n* = 46, 83.6%), followed by cleft palate (*n* = 5, 9.1%) and cleft lip (*n* = 4, 7.3%). No other congenital abnormalities were found in any of the children (*n* = 55, 100%). A family history of similar conditions was reported by 7 respondents (12.7%). Most children (*n* = 42, 76.4%) were diagnosed postnatally, whereas the rest (*n* = 13, 23.6%) were diagnosed prenatally.

**Table 2:** Characteristics of children with orofacial clefts (*n*=55)

Variable	Number (*n*)	Precentage (%)
Visits to YPPCBL/Cleft Center RSGM Unpad	11	20
One visit	4	7.3
Two visit	1	1.8
Three visit	39	70.9
More than three times		
Gender		
Male	36	65.5
Female	19	34.5
Age		
<12 months	9	16.4
1 year	2	3.6
2 year	7	12.7
3 year	12	21.8
4 year	13	23.6
5 year	9	16.4
6 year	2	3.6
7 year	1	1.8
Birth order		
1st	23	41.8
2nd	18	32.7
3rd	10	18.2
≥4th	3	5.5
NA	1	1.8
Type of orofacial cleft		
Cleft lip	4	7.3
Cleft palate	5	9.1
Cleft lip and palate	46	83.6
Syndromes and/or congenital abnormalities		
None	55	100
Time of orofacial cleft detection		
Prenatal	13	23.6
Postnatal	42	76.4
Family history		
Yes	7	12.7
No	47	85.5
NA	1	1.8

*NA* Not available

The results in Table 3 show that ANC services were received by 54 respondents (98.2%). The number of choices for each ANC provider (as respondents could choose more than one) was as follows: obstetricians (*n* = 25, 45.5%), general practitioners (*n* = 3, 5.5%), midwives (*n* = 46, 83.6%), and other healthcare providers (*n* = 1, 1.8%). Most ANC visits were conducted at healthcare facilities, such as doctor/midwife practices (*n* = 28, 50.9%).

**Table 3:** Antenatal care (ANC) history (*n*=55)

Variable	Number (*n*)	Precentage (%)
Received ANC services		
Yes	54	98.2
No	1	1.8
ANC service providers		
Obstetrician and Gynecologists	25	45.5
General Practitioner	3	5.5
Midwife	46	83.6
Other health workers	1	1.8
Did not receive ANC	1	1.8
ANC service locations		
Hospital	7	12.7
Maternity hospital/clinic	13	23.6
Public health center (*Puskesmas*)	6	10.9
Doctor/Midwife practice	28	50.9
Did not receive ANC	1	1.8
Proportion of ANC Access		
First trimester	47	85.5
Second trimester	7	12.7
Did not receive ANC	1	1.8
Proportion of ANC K4 (n=25)		
Adequate	22	88
Inadequate	3	12
Proportion of ANC K6 (n=30)		
Adequate	6	20
Inadequate	23	76.7
Did not receive ANC	1	3.3
Pregnancy awareness		
First trimester	50	90.9
Second trimester	2	3.6
NA	3	5.5
Ultrasound (USG) examination		
First trimester	22	40
Second trimester	29	52.7
Third trimester	43	78.2
Never	2	3.6
Ultrasound providers		
Obstetrician and Gynecologists	50	90.9
General Practitioner	2	3.6
Midwife	1	1.8
Did not perform ultrasound	2	3.6
Weighing and measuring height		
Yes	43	78.2
No	12	21.8
Blood pressure measurement		
Yes	53	96.4
No	2	3.6
Upper arm circumference (*LiLA*)		
Yes	42	76.4
No	13	23.6
Fundal height (*TFU*)		
Yes	43	78.2
No	12	21.8
Fetal heartbeat and presentation		
Yes	53	96.4
No	2	3.6
Tetanus immunization		
Yes	39	70.9
No	16	29.1
Iron tablet administration		
Yes	52	94.5
No	3	5.5
Laboratory tests		
Standard	12	21.8
Nonstandard	36	65.5
Don’t remember/don’t know	7	12.7
Case management by healthcare		
Yes	50	90.9
No	5	9.1
Counseling services		
Standard	3	5.5
Nonstandard	52	94.5
Integrated ANC 10T standards		
Complete	19	34.5
Incomplete	36	65.5

*NA* Not available

The timing of the first ANC visit (first contact) varied among respondents, but the majority attended during the first trimester (*n* = 47, 85.5%), whereas the remaining (*n* = 7, 12.7%) began in the second trimester. On the basis of the Ministry of Health regulations in effect during pregnancy, ANC K4 was completed by 22 out of 25 respondents (88%), whereas ANC K6, which requires at least one contact with a doctor during the first and third trimesters, was completed by 6 out of 30 respondents (20%). Most respondents who attended ANC were aware of their pregnancies from the first trimester (*n* = 50, 90.9%), particularly between the 2nd and 4th weeks of pregnancy.

Ultrasound (USG) examinations during pregnancy varied by trimester: 22 out of 55 pregnant women (40%) in the first trimester, 29 out of 55 pregnant women (52.7%) in the second trimester, and 43 out of 55 pregnant women (78.2%) in the third trimester. Additionally, two respondents reported never undergoing an ultrasound during their pregnancy. Ultrasound examinations were performed primarily by obstetricians (*n* = 50, 90.9%), followed by general practitioners (*n* = 2, 3.6%) and midwives (*n* = 1, 1.8%).

Table 4 presents the childbirth history of mothers after their pregnancies. Most mothers gave birth at midwife practices (*n* = 17, 30.9%), followed by government hospitals (*n* = 15, 27.3%) and private hospitals (*n* = 12, 21.8%). The majority of deliveries were assisted by obstetricians (*n* = 27, 49.1%) or midwives (*n* = 26, 47.3%). However, four (7.3%) of mothers delivered outside healthcare facilities, and two (3.6%) delivered without the assistance of healthcare professionals.

**Table 4:** Childbirth history (n=55)

Variable	Number (n)	Precentage (%)
Delivery location		
Government-funded public hospital	15	27.3
Private hospital	12	21.8
Clinic	5	9.1
Community health center	2	3.6
Midwife’s private practice	17	30.9
Home	4	7.3
Delivery assistance		
Obstetrician and Gynecologists	27	49.1
Midwife	26	47.3
Traditional birth attendant	1	1.8
Others	1	1.8
Source of payment		
BPJS/KIS (national health insurance)	29	52.7
Office funding	2	3.6
Self-funding	23	41.8
* Jampersal*	1	1.8
Delivery method		
Normal	37	67.3
Cesarean section	18	32.7
Delivery complication		
Transverse or breech position	3	5.5
Bleeding	3	5.5
Premature rupture of membranes (PROM)	4	7.3
Prolonged labor (>24 hours)	2	3.6
Umbilical cord loop	1	1.8
Placenta previa	1	1.8
Hypertension	3	5.5
Post-term pregnancy	1	1.8
No complications	38	69.1
Others	5	9.1
First aid efforts		
Immediate	15	27.2
Not immediate	2	3.6
No complications	38	69.1
Newborn physical examination		
Yes	45	81.8
No	10	18.2
Timing of newborn physical examination		
After birth when the baby is stable (within the first 6 hours)	34	61.8
6–48 hours after birth	3	5.5
3–7 days after birth	5	9.1
8–28 days after birth	3	5.5
Unknown	3	5.5
Not examined	7	12.7
Newborn physical examiner		
Pediatrician	14	25.5
Obstetrician and Gynecologists	10	18.2
Midwife	20	36.4
Neonatal nurse	4	7.3
Not examined	6	10.9
NA	1	1.8

*NA* Not available

The primary source of funding for childbirth was BPJS/KIS (*n* = 29, 52.7%), followed by private expenses (*n* = 23, 41.8%). A total of 37 mothers (67.3%) gave birth vaginally, while the remaining 18 mothers (32.7%) underwent a cesarean section. Approximately 17 mothers (30.9%) experienced at least one delivery complication, including premature rupture of membranes (*n* = 4, 7.3%), breech presentation, bleeding, or hypertension (*n* = 3, 5.5%), prolonged labor (*n* = 2, 3.6%), umbilical cord entanglement, placenta previa, postterm pregnancy (*n* = 1, 1.8%), and other complications, such as no contractions (*n* = 2, 3.6%), prolonged contractions, placental calcification, and cloudy amniotic fluid (*n* = 1, 1.8%). The majority (*n* = 15, 27.2%) sought immediate medical assistance, whereas the remaining (*n* = 2, 3.6%) did not seek help promptly.

Physical examinations of the newborns were performed on 45 babies (81.8%), whereas 10 babies (18.2%) did not receive such examinations. The timing of the examinations varied, with 34 babies (61.8%) examined immediately after birth, 3 babies (5.5%) within 6–48 h, 5 babies (9.1%) within 3–7 days, and 3 babies (5.5%) within 8–28 days after birth. Among the 10 babies who did not undergo a physical examination, 3 (5.5%) of parents were unaware of the timing, and 7 (12.7%) of babies were not examined. Physical examinations of newborns were conducted by healthcare professionals, including pediatricians (*n* = 14, 25.5%), obstetricians (*n* = 10, 18,2%), midwives (*n* = 20, 36.4%), and neonatal nurses (*n* = 4, 7.3%).

## Discussion

This study explores the experiences of pregnant mothers who gave birth to children with orofacial clefts, focusing on their ANC practices and the delivery process they underwent. This study aims to explore mothers’ personal experiences during pregnancy without examining causal relationships between ANC and the birth of children with abnormalities.

The characteristics of parents of children with orofacial clefts include aspects related to education, occupation, and geographical distribution. Most mothers are within the ideal reproductive age range of 20–35 years, which is often associated with lower pregnancy risks (Table 1). Research by Andriani et al. highlights a significant relationship between maternal age, adherence to ANC, and childbirth assistance by healthcare professionals [31].

Nearly half of the fathers and mothers had attained the secondary level of education (Table 1), potentially fostering greater health awareness within the household. A higher maternal education level is associated with a greater likelihood of delivering in healthcare facilities, while the father’s education also plays a critical role in utilizing healthcare services. This finding indicates that the educational attainment of both parents can influence family health decisions. Additionally, most fathers are employed, actively contributing to the family economy, which may directly impact their ability to access healthcare services [32].

In terms of employment, the majority of fathers (*n* = 51, 92.7%) are employed, whereas most mothers (*n* = 41, 74.5%) are unemployed (Table 1). This reflects traditional roles where fathers are the primary breadwinners, whereas mothers focus more on household responsibilities. However, maternal employment status also plays a role in determining the utilization of postnatal healthcare services. Research indicates that employed mothers tend to be more financially independent, enabling them to access healthcare services more effectively than nonworking mothers do [32].

Geographically, the majority of the respondents reside in urban areas (*n* = 44, 80%), whereas the remaining respondents live in rural areas (*n* = 11, 20%) (Table 1). This distribution indicates that most respondents have closer access to healthcare facilities, which are generally more abundant in urban areas. However, families living in rural areas are likely to face greater challenges in accessing healthcare facilities than families in urban areas are [31, 32]. Therefore, it is important to consider that while parental education and employment provide a strong foundation for understanding access to healthcare services, the location of residence remains a factor that needs further evaluation.

The results of this study revealed that the majority of orofacial cleft cases occurred in male infants, with 36 cases (65.5%), whereas female infants accounted for only 19 cases (34.5%) (Table 2). This is consistent with the findings of the study by da Silva, Calumby, and Freitas, who reported that orofacial clefts were most commonly observed in male infants, accounting for 58.8% of all cases [33].

The combination of cleft lip and palate was the most common type among the children, with 46 cases (83.6%) (Table 2). This finding aligns with the findings of Salari et al., who examined the global prevalence of orofacial clefts. The highest prevalence was found for the combination of cleft lip and palate, at 0.45 per 1,000 live births, followed by cleft palate at 0.33 and cleft lip at 0.3 per 1,000 live births [7].

Several previous studies have shown that the risk of orofacial clefts is greater in children with higher birth orders. Research by Acuña-González et al. and Cheshmi et al. revealed that third- or fourth-born children and those born later had a greater risk of experiencing orofacial clefts [19, 34]. However, in this study, we found that most children with orofacial clefts were firstborn (*n* = 23, 41.8%) (Table 2). This result is consistent with the findings of Noorollahian et al., who also reported that first-born children were more frequently affected by orofacial clefts, especially in regions with a high prevalence of consanguineous marriage [35]. Consanguineous marriage, typically defined as marriage between first cousins or closer relatives, either from the father’s or mother’s side, may contribute to greater genetic risk. Since data on consanguineous marriage were not collected in this study, this factor cannot be confirmed as the primary cause. Other factors, such as maternal age, socioeconomic conditions, and nutritional habits during pregnancy, may also influence these difference [19].

Nonsyndromic orofacial clefts are a type of isolated orofacial cleft that is not associated with other anomalies. Approximately 70% of cases of cleft lip and/or cleft palate are of this type, whereas the syndromic type is relatively rare [10]. This is consistent with our findings, where all the children in our study (*n* = 55, 100%) did not have syndromes or other congenital abnormalities (Table 2). The combination of genetic and environmental factors, such as folic acid deficiency, smoking, alcohol consumption, and infections, plays a significant role in the pathogenesis of nonsyndromic cleft lip and palate in susceptible individuals [10].

Prenatal diagnosis of orofacial clefts was found in 13 children (23.6%), whereas 42 children (76.4%) were diagnosed postnatally (Table 2). These data indicate that prenatal diagnosis of orofacial clefts remains relatively rare. Most orofacial cleft cases are identified postnatally, as noted in the study by Farladansky-Gershnabel et al., which reported that only 20–30% of cases are diagnosed prenatally [36]. However, a more recent study by Sander et al. revealed an increase in prenatal detection for cleft lip with or without palate, reaching 71.7% in Denmark, although the prenatal detection rate for cleft palate alone was only 3.3% (*p* < 0.0001) [17]. The posterior palate is often difficult to visualize because of the acoustic shadow of the facial bones [12]. Even in the study by Hanny et al., many cases were detected more than 30 days postnatally [14].

Ultrasonography (US) remains the primary standard for prenatal diagnosis of orofacial clefts, although it has limitations [12]. These limitations may be due to fetal positioning, limited US resolution, and the experience of the sonographer [37, 38]. The accuracy of ultrasonographic detection, particularly for cleft palate (CPO), tends to be low because of difficulties in visualization during US examination [12, 15]. Many orofacial cleft cases are diagnosed only after birth, which can lead to delayed medical intervention and increased emotional stress for the family [14, 36]. For a more accurate diagnosis, a combination of 2D and 3D ultrasound has been shown to significantly improve accuracy [2, 12]. In some cases, fetal magnetic resonance imaging (MRI) is used as an additional examination to obtain clearer visualization [15, 37]. Adequate training for sonographers and access to better equipment can help with earlier detection [24, 38]. Counseling for parents and early treatment planning also depend on the quality of prenatal diagnosis [16]. The ideal time for the most accurate examination to detect orofacial clefts is during the second trimester [17]. However, among the three trimesters, most respondents performed their ultrasound examinations most frequently during the third trimester, with 43 respondents (78.2%) selecting this period (Table 3). Additionally, two mothers did not undergo ultrasound during pregnancy (Table 3). The lack of ultrasound examination may be due to limited access to ultrasound equipment and insufficient training for sonographers [39].

Most mothers in this study attended antenatal clinics, which is known to positively impact both maternal health and newborn care. WHO emphasizes that regular ANC visits enable timely identification and management of pregnancy-related complications, improve maternal nutritional status, and enhance preparedness for childbirth and newborn care, ultimately improving pregnancy outcomes and reducing the risk of maternal and neonatal complications [6]. In this study, the benefits of ANC are reflected in the high coverage of at least one ANC visit among respondents (98.2%), which aligns with the 2023 Indonesian Health Survey (SKI) report (98.4%). ANC services in Indonesia include counseling on nutrition, hygiene, recognition of pregnancy danger signs, birth preparedness planning, and newborn care such as early initiation of breastfeeding, proper cord care, and detection of neonatal danger signs. In the case of congenital anomalies such as orofacial cleft, ANC also facilitates early diagnosis, provides parental guidance, and enables planning for feeding strategies, psychosocial support, and surgical referral [4].

These observations are consistent with evidence from a systematic review showing that antenatal education interventions improve maternal confidence, reduce anxiety, and promote better neonatal care practices, contributing to improved maternal and newborn outcomes [40]. Despite this, variation in ANC visit standards was observed: among mothers pregnant before 2021, 22 out of 25 mothers (88%) met the ANC K4 standard, whereas only 6 out of 30 mothers (20%) of those pregnant after 2021 met the ANC K6 standard (Table 3). The low compliance with the ANC K6 standard reflects challenges in adopting the new policy, one of which is its relatively recent implementation. Low adherence to the ANC K6 standard may have important implications for maternal and neonatal outcomes, as it can reduce opportunities for comprehensive assessments, preventive interventions such as tetanus toxoid immunization and iron/folate supplementation, and maternal education on birth preparedness and newborn care. In the case of orofacial cleft, these missed opportunities can delay diagnosis, limit preparation for specialized care, and potentially increase neonatal risks. These gaps may also delay the detection and management of pregnancy complications and increase the risk of adverse neonatal outcomes, including low birth weight, infections, and mortality [6, 29, 40].

Additionally, an age-based analysis revealed that out of the 28 mothers who adhered to either the ANC K4 or ANC K6 standards (depending on the applicable policy), 24 (85.7%) were in the 20–35 age group (Tables 1 and 3). This finding aligns with research by Nurmawati and Indrawati, who reported that pregnant women in this age group tend to visit ANC visits more consistently than do those under 20 years of age, who may be less prepared, and those over 35 years of age, who often feel that they have sufficient experience [22].

Most of the respondents’ children in this study were 3 and 4 years old, with 12 children (21.8%) and 13 children (23.6%), respectively (Table 2). These findings indicate that their pregnancies occurred during the COVID-19 pandemic, which began in 2020. The pandemic has posed various challenges for pregnant women accessing ANC services, resulting in decreased adherence to ANC visits. This decline was due to concerns about infection risks, mobility restrictions during lockdowns, and the reallocation of healthcare facilities to handle COVID-19 cases [23]. Other studies have highlighted additional factors, such as pregnant women’s knowledge of ANC and economic constraints. Therefore, it is crucial to consider these factors when evaluating the performance of ANC services and ensuring compliance with service standards [41].

In addition to the number of visits, this study also revealed that the coverage of integrated ANC 10T services remains very low. Only 19 pregnant women (34.5%) received services meeting the standard. One of the services with the lowest coverage was laboratory testing, which was received by only 21.8% of pregnant women. Most ANC check-ups were conducted at doctor or midwife practices (50.9%), with the majority of services provided by midwives (83.6%) (Table 3). These findings align with those of previous studies, which reported low coverage of comprehensive ANC 10T services for pregnant women [42]. Factors contributing to this low coverage include limited infrastructure and resources at healthcare facilities, such as insufficient equipment and staff to support standard ANC services. The competence of healthcare workers, particularly midwives, also poses a significant challenge, especially if they lack adequate training or knowledge of ANC guidelines [42].

The coverage of deliveries assisted by healthcare professionals was excellent, with most respondents (51 or 92.7%) giving birth at healthcare facilities and 53 (96.4%) assisted by skilled birth attendants (Table 4). However, 4 respondents (7.3%) gave birth outside healthcare facilities, and 2 (3.6%) were assisted by non-health professionals. A total of 17 respondents (30.9%) delivered at midwife practices, with BPJS/KIS being the primary source of financing for 29 respondents (52.7%) (Table 4). These numbers highlight the role of midwife practices and BPJS/KIS in facilitating access to maternity services at healthcare facilities. A total of 37 respondents (67.3%) experienced normal deliveries. Additionally, 17 respondents (30.9%) experienced complications during delivery, with most seeking immediate assistance (Table 4). This emphasizes the importance of education and preparedness in managing delivery complications.

A total of 21 out of 55 babies (38.2%) were identified as having orofacial clefts several hours to days after birth during neonatal visits (Table 4). This delay is likely due to insufficiently thorough newborn physical examinations, particularly suboptimal palpation and visualization of the mouth [43]. If initial physical examinations are missed or conducted inadequately, congenital abnormalities such as orofacial clefts may be undetected. Research by Hanny et al. also indicated that the more severe the orofacial cleft or the presence of other congenital abnormalities, the easier it is to diagnose earlier [14]. Thus, comprehensive physical examinations are essential for detecting congenital abnormalities and ensuring timely interventions.

This study highlights the need to improve healthcare access by providing equipment such as ultrasound technology and comprehensive training for sonographers to increase the accuracy of prenatal anomaly detection. Additionally, medical training can be enhanced to ensure that newborn physical examinations are conducted thoroughly, enabling the early detection of abnormalities such as orofacial clefts. These findings are expected to serve as a preliminary reference to guide efforts in improving the quality of maternal and newborn healthcare services, particularly in optimizing the early detection of congenital anomalies.

This study has some limitations. This study was limited to patients attending YPPCBL, the majority of whom belong to lower-middle economic groups. As a result, other economic classes may have been underrepresented, which could influence the diversity of the study’s findings. In a few cases, when mothers were unavailable to participate due to work or other constraints, responses were obtained from the father or a close relative. Although this occurred rarely (only one father and one relative), it may have affected the consistency of perspectives regarding antenatal care. Future research should aim to include a more diverse population and ensure direct participation of mothers to provide a more comprehensive and consistent perspective.

## Conclusions

This study indicates that although most mothers of children with orofacial clefts have received antenatal care (ANC), its implementation still has shortcomings, such as low compliance with ANC K6 visits and services that do not fully meet the 10T integrated ANC standards. Prenatal detection of orofacial clefts remains rare. Furthermore, while most deliveries occur in healthcare facilities, some cases, such as palatal clefts, remain difficult to detect at birth owing to inadequate attention during newborn physical examination. This highlights the need for improvements in the quality of ANC services and neonatal examinations to detect abnormalities early.

## Supplementary Information

Supplementary Material 1.

## Data Availability

No data are available due to privacy restrictions.

## Abbreviations

ANC: Antenatal Care
NICU: Neonatal Intensive Care Unit
USG: Ultrasonography
CPO: Cleft Palate Only
CL/P: Cleft Lip and Palate
K1: First antenatal care visit
K4: Fourth antenatal care visit
K6: Sixth antenatal care visit
BPJS: Badan Penyelenggara Jaminan Sosial (social security provider)
KIS: Kartu Indonesia Sehat (Health Insurance Card)
SKI: Survei Kesehatan Indonesia (Indonesian Health Survey)

## References

[1] Aprilia W. Perkembangan pada masa pranatal dan kelahiran. Yaa Bunayya: Jurnal Pendidikan Anak Usia Dini. 2020;4(1):39–55.

[2] Loomis-Goltl E, Briley P, Kotlarek KJ. Impact of Prenatal Care on Newborn Complications for Infants with Cleft Lip with or Without Cleft Palate. Cleft Palate Craniofac J. 2024;61(6). 10.1177/10556656231153453. 10.1177/10556656231153453 PMC10387128 36718491

[3] World Bank Group. UNDESA. Trends in maternal mortality estimates 2000 to 2023. Report. 2025. https://www.who.int/publications/i/item/9789240108462

[4] Kemenkes RI. Pedoman pelayanan antenatal terpadu edisi ketiga. Jakarta: Kementerian Kesehatan Republik Indonesia; 2020.

[5] United Nations Inter-agency Group for Child Mortality Estimation (UN IGME). Levels and Trends in Child Mortality: Report 2024. New York. 2024. Report. Available from: https://data.unicef.org/resources/levels-and-trends-in-child-mortality-2024/. [cited 2025 Aug 5].

[6] World Health Organization. Recommendations in antenatal care for a positive pregnancy experience. 2016. https://www.who.int/publications/i/item/9789241549912 28079998

[7] Salari N, Darvishi N, Heydari M, Bokaee S, Darvishi F, Mohammadi M. Global prevalence of cleft palate, cleft lip and cleft palate and lip: A comprehensive systematic review and meta-analysis. J Stomatol Oral Maxillofac Surg. 2022;123(2):110–20. 10.1016/j.jormas.2021.05. .008 PubMed PMID: 34033944. 34033944 10.1016/j.jormas.2021.05.008

[8] Rachmawati AI, Puspitasari RD, Cania E. Faktor-Faktor yang Memengaruhi Kunjungan Antenatal Care (ANC) Ibu Hamil. Majority: Medical Journal of Lampung University. 2017;7(November):72–6. Available from: https://juke.kedokteran.unila.ac.id/index.php/majority/article/view/1748.

[9] World Health Organization. Global Registry and Database on Craniofacial Anomalies. Geneva: World Health Organization; 2003.

[10] Babai A, Irving M. Orofacial Clefts: Genetics of Cleft Lip and Palate. Genes (Basel). 2023;14(8):1603. 10. 3390/genes14081603 PubMed PMID: 37628654. 37628654 10.3390/genes14081603 PMC10454293

[11] Kemenkes RI. Badan Kebijakan Pembangunan Kesehatan. Survei Kesehatan Indonesia (SKI) 2023. Report. 2023. https://www.badankebijakan.kemkes.go.id/hasil-ski-2023/

[12] Dabadie A, Quarello E, Degardin N, Desbriere R, Heckenroth H, Sigaudy S, et al. Added Value of MRI for the Prenatal Diagnosis of Isolated Orofacial Clefts and Comparison with Ultrasound. Diagn Interv Imaging. 2016;97(9):915–21. .015 PubMed PMID: 26969118. 26969118 10.1016/j.diii.2015.11.015

[13] Farladansky-Gershnabel S, Gluska H, Halevi N, Kotser N, Sharon-Weiner M, Schreiber H, et al. Cleft Lip and/or Cleft Palate: Prenatal Accuracy, Postnatal Course, and Long-Term Outcomes. Children. 2022;9(12). 10.3390/children9121880. 10.3390/children9121880 PMC9776564 36553322

[14] Hanny KH, de Vries IAC, Haverkamp SJ, Oomen KPQ, Penris WM, Eijkemans MJC, et al. Late Detection of Cleft Palate. Eur J Pediatr. 2016;175(1):71–80. 10.1007/s00431-015-2590-9. 26231683 10.1007/s00431-015-2590-9 PMC4709386

[15] Gai S, Wang L, Zheng W. Comparison of prenatal ultrasound with MRI in the evaluation and prediction of fetal orofacial clefts. BMC Med Imaging. 2022;22(1):213. 10.1186/s12880-022-00929-9. 36471263 10.1186/s12880-022-00929-9 PMC9720929

[16] Sreejith V, Arun V, Devarajan A, Gopinath A, Sunil M. Psychological Effect of Prenatal Diagnosis of Cleft Lip and Palate: A Systematic Review. Contemp Clin Dent. 2018;9(2):304–8. 10.4103/ccd.ccd_673_17. 29875578 10.4103/ccd.ccd_673_17 PMC5968700

[17] Sander FH, Jørgensen DS, Jakobsen LP, Jensen AN, Lousen T, Sandager P, et al. Prenatal Detection of Orofacial Clefts in Denmark From 2009 to 2018. Ultrasound Obstet Gynecol. 2024;63(4):507–13. 10. 1002/UOG.27488 PubMed PMID: 37724632. 37724632 10.1002/uog.27488

[18] Efendi F, Ni’Mah AR, Hadisuyatmana S, Kuswanto H, Lindayani L, Berliana SM. Determinants of facility-based childbirth in Indonesia. Sci World J. 2019;2019(1):9694602. 10.1155/2019/9694602 . PubMed PMID: 31320842. 10.1155/2019/9694602 PMC6610729 31320842

[19] Acuña-González G, Medina-Solís CE, Maupomé G, Escoffié-Ramírez M, Hernández-Romano J, Marquez-Corona L, et al. Family history and socioeconomic risk factors for non-syndromic cleft lip and palate: A matched case-control study in a less developed country. Biomedica. 2011;31:381–91. 10.1590/S0120-41572011000300010. 22674314 10.1590/S0120-41572011000300010

[20] Vieira AR, Orioli IM. Birth Order and Oral Clefts: A Meta Analysis. Teratology. 2002;66(5):209–16. 10.1002/tera.10088. 12397628 10.1002/tera.10088

[21] Ocktariyana O, Rohmah UN, Yulia S, Rosnani R, Mediarti D, Setyowati S, et al. Antenatal Care in Indonesia: A Nationwide Study. Br J Midwifery. 2023;31(10). 10.12968/bjom.2023.31.10.558.

[22] Nurmawati IF. Cakupan Kunjungan Antenatal Care pada Ibu Hamil. Higeia: J Public Health Res Dev. 2018;2(1):113–24.

[23] Ariani N. Antenatal Care Services Utilization During COVID-19 Second Wave Attack in Pasuruan, Indonesia. J Med Life. 2022;15(1). 10.25122/jml-2021-0238. 10.25122/jml-2021-0238 PMC8852645 35186130

[24] Olusanya A, Michael A, Olawoye O, Akinmoladun V. Antenatal Events Amongst Mothers of Babies with Orofacial Clefts. Ann Ib Postgrad Med. 2020;18:S2–8. 33071689 PMC7513381

[25] Ramos AM, Marceau K, Neiderhiser JM, De Araujo-Greecher M, Natsuaki MN, Leve LD. Maternal Consistency in Recalling Prenatal Experiences at 6 Months and 8 Years Postnatal HHS Public Access. J Dev Behav Pediatr. 2020;41(9):698–705. 10.1097/DBP.0000000000000841. 32740284 10.1097/DBP.0000000000000841 PMC7942020

[26] Sugiyono. Metode Penelitian Kuantitatif, Kualitatif, dan R&D. Metode Penelitian dan Pengembangan Pendekatan Kualitatif. Kuantitatif, dan R&D. Bandung: Penerbit Alfabeta; 2022.

[27] Yusoff MSB, ABC of Content Validation and Content Validity Index Calculation. Educ Med J. 2019;11(2). 10.21315/eimj2019.11.2.6.

[28] Budiastuti D, Bandur A. Validitas dan Reliabilitas Penelitian. Jakarta: Mitra Wacana Media; 2018.

[29] Menteri Kesehatan RI. Peraturan Menteri Kesehatan Republik Indonesia Nomor 21 Tahun 2021 tentang Penyelenggaraan Pelayanan Kesehatan Masa Sebelum Hamil, Masa Hamil, Persalinan, dan Masa Sesudah Melahirkan, Pelayanan Kontrasepsi, dan Pelayanan Kesehatan Seksual. Kementrian Kesehatan Republik Indonesia; 2021.

[30] Badan Pusat Statistik (BPS). Peraturan Kepala Badan Pusat Statistik Nomor 120 Tahun 2020 tentang Klasifikasi Desa Perkotaan dan Perdesaan di Indonesia 2020: Buku 2 Jawa. 2020. Jakarta: Badan Pusat Statistik; 2020. 446.

[31] Andriani H, Rachmadani SD, Natasha V, Saptari A. Continuity of maternal healthcare services utilisation in Indonesia: Analysis of determinants from the Indonesia Demographic and Health Survey. Fam Med Community Health. 2021;9(4). 10.1136/fmch-2021-001389. 10.1136/fmch-2021-001389 PMC8710424 34937797

[32] Aji RS, Efendi F, Kurnia ID, Tonapa SI, Chan CM. Determinants of maternal healthcare service utilisation among Indonesian mothers: A population-based study. F1000Res. 2021;10:1124. 10.12688/f1000research.73847.2. 35602669 10.12688/f1000research.73847.1 PMC9086521

[33] da Silva AM, Calumby RT, Freitas VS. Epidemiologic Profile and Prevalence of Live Births with Orofacial Cleft in Brazil: A Descriptive Study. Revista Paulista de Pediatria. 2024;42. 10.1590/1984-0462/2024/42/2022234 . PubMed PMID: 37729242. 10.1590/1984-0462/2024/42/2022234 PMC10508041 37729242

[34] Cheshmi B, Jafari Z, Naseri MA, Davari HA. Assessment of the Correlation Between Various Risk Factors and Orofacial Cleft Disorder Spectrum: A Retrospective Case-Control Study. Maxillofac Plast Reconstr Surg. 2020;42(1). 10.1186/s40902-020-00270-7. 10.1186/s40902-020-00270-7 PMC7415041 32802820

[35] Noorollahian M, Nematy M, Dolatian A, Ghesmati H, Akhlaghi S, Reza Khademi G. Cleft Lip and Palate and Related Factors: A 10 Years Study in University Hospitalised Patients at Mashhad-Iran. 2015. 10.4103/0189-6725.172576. 10.4103/0189-6725.172576 PMC4955481 26712297

[36] Farladansky-Gershnabel S, Gluska H, Halevi N, Kotser N, Sharon-Weiner M, Schreiber H, et al. Cleft Lip and/or Cleft Palate: Prenatal Accuracy, Postnatal Course, and Long-Term Outcomes. Children. 2022;9(12). 10.3390/children9121880. 10.3390/children9121880 PMC9776564 36553322

[37] Baeza-Pagador A, Tejero-Martínez A, Salom-Alonso L, Camañes-Gonzalvo S, García-Sanz V, Paredes-Gallardo V. Diagnostic Methods for the Prenatal Detection of Cleft Lip and Palate: A Systematic Review. J Clin Med. 2024;13(7). 10.3390/JCM13072090/S1. 10.3390/jcm13072090 PMC11012824 38610855

[38] Maarse W, Pistorius LR, Van Eeten WK, Breugem CC, Kon M, Van Den Boogaard MJH, et al. Prenatal Ultrasound Screening for Orofacial Clefts. Ultrasound Obstet Gynecol. 2011;38(4):434–9. 10. 1002/UOG.8895 PubMed PMID: 21113916. 21113916 10.1002/uog.8895

[39] Ginsburg AS, Liddy Z, Khazaneh PT, May S, Pervaiz F. A Survey of Barriers and Facilitators to Ultrasound Use in Low- and Middle-Income Countries. Sci Rep. 2023;13(1). 10.1038/s41598-023-30454-w. 10.1038/s41598-023-30454-w PMC9969046 36849625

[40] Hong K, Hwang H, Han H, Chae J, Choi J, Jeong Y, et al. Perspectives on antenatal education associated with pregnancy outcomes: Systematic review and meta-analysis. Women Birth. 2021. 10.1016/j.wombi.2020.04.002. 32360106 10.1016/j.wombi.2020.04.002

[41] Anggraini Lolitasari, Tendean HMM, Suparman E. Gambaran Pelayanan Antenatal pada Masa Pandemi COVID-19 di Indonesia 2020–2021. e-CliniC. 2023;11(3). 10.35790/ecl.v11i3.45182.

[42] Harianis S, Ritasari M, Asrita Sari DE, Madinah M. Analisis Faktor Pelayanan Antenatal Terpadu Di Wilayah Kerja Puskesmas Tembilahan Hulu. JOMIS (Journal Midwifery Science). 2020;4(1). 10.36341/jomis.v4i1.1078.

[43] Kementerian Kesehatan RI. Peraturan Menteri Kesehatan Republik Indonesia Nomor 53 Tahun 2014 tentang Pelayanan Kesehatan Neonatal Esensial. Kementrian Kesehatan RI, Jakarta. Indonesia; 2014.

