# Supplementary material for: Antenatal care and childbirth experience among parents of children with orofacial clefts: an observational study
Source: BMC Oral Health. 2026 May 13;26:1218. doi: 10.1186/s12903-026-08513-1 (PMC13348264; doi:10.1186/s12903-026-08513-1)
Supplement: Supplementary file 1 — Supplementary Material 1. [file 12903_2026_8513_MOESM1_ESM.docx]

**ANTENATAL CARE AND CHILDBIRTH EXPERIENCE**

**AMONG PARENTS OF CHILDREN WITH OROFACIAL CLEFTS**

Please place a check mark (✔) on the appropriate answer choice.

| **Parent Characteristics** | | |
| --- | --- | --- |
| Full name: | | |
| Contact number: | | |
| Who filled out this questionnaire?  🗆 The mother  🗆 The father  🗆 Others, please mention here…….. | | |
| How old was Mom when the pregnancy took place?  🗆 <20 years  🗆 20–35 years  🗆 >35 years | | |
| What is the latest education that Mom have undergone?  🗆 *SD/MI Sederajat* (elementary school)  🗆 *SMP/MTs/Sederajat* (junior high school)  🗆 *SMA/SMK/MA Sederajat* (senior high school)  🗆 *AKADEMI/DI/DII/DIII* (Academy/ Diploma I/Diploma II/Diploma III)  🗆 UNIVERSITAS/DIPLOMA IV/SARJANA (University/ Diploma IV/ Undergraduate) | | |
| What is the latest education that Mom have undergone?  🗆 SD/MI Sederajat (elementary school)  🗆 SMP/MTs/Sederajat (junior high school)  🗆 SMA/SMK/MA Sederajat (senior high school)  🗆 AKADEMI/DI/DII/DIII (Academy/ Diploma I/Diploma II/Diploma III)  🗆 UNIVERSITAS/DIPLOMA IV/SARJANA (University/ Diploma IV/ Undergraduate) | | |
| Parents occupation  **Father**  🗆 Employed  🗆 Unemployed  **Ibu**  🗆 Employed  🗆 Unemployed | | |
| Where do you reside?  🗆 Urban  🗆 Rural | | |
|  | | |
| **Child characteristics** | | |
| Gender  🗆 Boy  🗆 Girl | | |
| How old is the child? | | |
| 🗆 <12 months  🗆 1 year  🗆 2 years | 🗆 3 years  🗆 4 years  🗆 5 years | 🗆 6 years  🗆 7 years  🗆 8 years |
| Which child is this?  🗆 first  🗆 second  🗆 third  🗆 fourth or more | | |
| What type is the orofacial cleft of your child?  🗆 Cleft lip  🗆 Cleft palate  🗆 Cleft lip and palate | | |
| Does your child have syndromes and/ or congenital abnormalities?  🗆 Yes  🗆 No | | |
| When did you find out about your child's cleft lip and/ or palate condition?  🗆 Before childbirth  🗆 After childbirth | | |
| Does anyone in your family have a cleft lip or palate?  🗆 Yes  🗆 No | | |
| How many visits to the YPPCBL/Cleft Center at RSGM UNPAD has your child made??  🗆 One time  🗆 Two times  🗆 Three times  🗆 More than three times | | |
| **Antenatal Care Experience** | | |
| 1. When you were pregnant, did you have a prenatal check-up?   🗆 Yes  🗆 No  *** *If no, go to question 25 about childbirth experience.* | | |
| 1. During pregnancy, where did you go for pregnancy check-ups?   (You can choose more than one answer):  🗆 Obstetrician  🗆 General practicioner  🗆 Midwife  🗆 Other’s health workers | | |
| 1. Where do you most often go for gynecological check-ups?   🗆 Public/ private hospital  🗆 Maternity hospital/ clinic  🗆 Public health center (*Puskesmas*)  🗆 *Puskesmas pembantu*  🗆 Doctor/ Midwife practice  🗆 *Poskesdes/Polindes*  🗆 *Posyandu*  🗆 At home | | |
| 1. When did you first check your pregnancy? (There are respondents who are more familiar with writing trimester information using weekly calculations)   🗆 Trimester I (1-12 weeks/ at first three months)  🗆 Trimester II (13-26 weeks/ between 4-6 months)  🗆 Trimester III (27-40 weeks/ between 7 months until labor) | | |
| 1. When did you first learn of the pregnancy? (please write down the week)   On week………... | | |
| 1. During the first trimester (1-12 weeks gestation), how many prenatal check-up visits were made?   🗆 One time  🗆 More than one time  🗆 Never | | |
| 1. During the second trimester (13-26 weeks gestation), how many prenatal check-up visits were made?   🗆 One time  🗆 Two times  🗆 More than two times  🗆 Never | | |
| 1. During the first trimester (27-40 weeks gestation), how many prenatal check-up visits were made?   🗆 One time  🗆 Two times  🗆 Three times  🗆 More than three times  🗆 Never | | |
| 1. During the first and third trimesters, did you have at least one pregnancy check-up with a doctor?   (You can choose more than one answer):  🗆 Yes, at first trimester  🗆 Yes, at third trimester  🗆 Never | | |
| 1. During pregnancy, how many times did the mother have an ultrasound examination?   (You can choose more than one answer):  🗆 Trimester I  🗆 Trimester II  🗆 Trimester III | | |
| 1. Who provides ultrasound examination services during pregnancy?   (You can choose more than one answer):  🗆 Dokter kandungan  🗆 Dokter umum | | |
| 1. At the time of the prenatal check-up, was your weight measured?   🗆 Yes  🗆 No | | |
| 1. At the time of the prenatal check-up, was your height measured?   🗆 Yes  🗆 No | | |
| 1. At the time of the prenatal check-up, was your blood pressure measured?   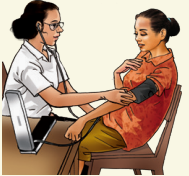  🗆 Yes  🗆 No | | |
| 1. At the time of the prenatal check-up, did your arm circumference measured (*LiLa*)?   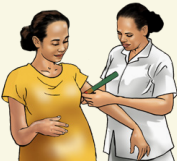  🗆 Yes  🗆 No | | |
| 1. During the pregnancy checkup, did your height of the uterus measured (fundus height)?   (Using a cloth meter)**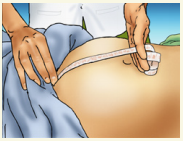**  🗆 Yes  🗆 No | | |
| 1. During the pregnancy check-up, did your abdomen examined (palpated) to determine the position of the fetus?   **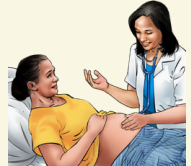**  🗆 Yes  🗆 No | | |
| 1. During the pregnancy check-up, did the fetal heart rate checked (*DJJ*)?   🗆 Yes  🗆 No | | |
| 1. At the time of pregnancy check-up, did you get immunization in the form of an injection in the upper arm to prevent the baby from tetanus disease?   🗆 Yes  🗆 No | | |
| 1. At the time of the antenatal check-up, was you given at least 90 blood supplement tablets during pregnancy?   🗆 Yes  🗆 No | | |
| 1. During the pregnancy check-up, did you have any laboratory tests?   **Select the form of examination** performed.  (You can choose more than one answer):  🗆 Pregnancy test  🗆 Hb test  🗆 Blood type test  🗆 HIV, syphilis, hepatitis B tests  🗆 Not remember/ Do not know  🗆 Never took any test  🗆 Others, please mention…….. | | |
| 1. During pregnancy check-ups, did you receive any health services to address your complaints? **Select the form of service** you received.   (You can choose more than one option):  🗆 Provide appropriate treatment  🗆 Refer to specialists if needed  🗆 Ensure pregnant women receive adequate care to maintain their health and the health of their fetus  🗆 Not performed | | |
| 1. During pregnancy check-ups, did you receive counseling from a health worker?   Select the form of counseling that you received.  (You can choose more than one option):  🗆 Inform the inspection/ examination result  🗆 Provide care according to gestational age and maternal age  🗆 Nutrition of pregnant women  🗆 Mental readiness  🗆 Recognizing pregnancy emergency signs  🗆 Childbirth  🗆 Postpartum  🗆 Childbirth preparation  🗆 Postpartum contraception  🗆 New born care  🗆 Early breastfeeding initiation  🗆 Exclusive breastfeeding  🗆 Not performed | | |
| 1. Are the above pregnancy checks (questions 12-23) done every visit?   🗆 Yes  🗆 No | | |
| **Childbirth Experience** | | |
| 1. Who helps during childbirth?   🗆 Obstetrician and gynecologist  🗆 General practicioner  🗆 Midwife  🗆 Nurse  🗆 Traditional birth attendant  🗆 No one  🗆 Others, please mention…….. | | |
| 1. Where is the location where you gave birth?   🗆 Public hospital  🗆 Private hospital  🗆 Clinic  🗆 Public Health Center or *Puskesmas/Pustu/Pusling*  🗆 Doctor practice  🗆 Midwife practice  🗆 *Poskesdes/Polindes*  🗆 Home  🗆 Others, please mention…….. | | |
| 1. What is the source of costs used when you gives birth?   🗆 *BPJS/KIS*  🗆 Private insurance  🗆 Office expenses  🗆 Other people’s costs  🗆 Out of pocket expenses  🗆 *Jampersal*  🗆 *Jamperda*  🗆 Others, please mention…….. | | |
| 1. What are the types of methods or ways when you give birth?   🗆 Normal  🗆 Caesarian section  🗆 Others, please mention…….. | | |
| 1. What health problems or disorders did you experience during childbirth?   🗆 Transverse/ inverse fetal position  🗆 Bleeding  🗆 Seizure  🗆 Premature/ early membranes rupture  🗆 Prolonged labor (>24 hours)  🗆 Umbilical cord twists  🗆 Placenta previa (baby's placenta covers part/all of the uterine mouth)  🗆 Retained placenta  🗆 Hipertention  🗆 Over-month pregnancy  🗆 No problem or disorder  🗆 Others, please mention…….. | | |
|  | | |
| 1. When experiencing the above disorders, when were first aid efforts by health workers given to the mother?   🗆 Immediately (< 30 minutes after get the complication)  🗆 Not immediately  🗆 No looking for first aid  🗆 No problem or disorder | | |
| 1. When your child was born, did a physical examination that included looking at the inside of the mouth (shape of the lips, gums, and palate) performed?   🗆 Yes  🗆 No | | |
| 1. When was the first complete physical examination of your baby performed? (including examination of the inside of the mouth, including the shape of the lips, gums, and palate)   🗆 After birth when the baby is stable (before 6 hours have passed)  🗆 6–48 hours after birth  🗆 3–7 days after birth  🗆 8–28 days after birth  🗆 Do not know  🗆 Never performed | | |
| 1. Who first conducted the examination?   🗆 Paediatric  🗆 Obstetric and gynecologist  🗆 General practicioner  🗆 Midwife  🗆 Neonatal nurse  🗆 Traditional birth attendant  🗆 Never performed | | |
